# Probe-assisted ILM-Rhexis: A Novel Peeling Technique

**DOI:** 10.18502/jovr.v21.18171

**Published:** 2026-06-19

**Authors:** Ramin Nourinia, Seyed-Hossein Abtahi, Hosein Nouri, Amir Reza Mansouri

**Affiliations:** ^1^Ophthalmic Research Center, Research Institute for Ophthalmology and Vision Science, Shahid Beheshti University of Medical Sciences, Tehran, Iran; ^2^Labbafinejad Medical Center, Shahid Beheshti University of Medical Sciences, Tehran, Iran; ^3^School of Medicine, Isfahan University of Medical Sciences, Isfahan, Iran; ^4^This manuscript has been submitted to Research Square as a preprint (DOI: https://doi.org/10.21203/rs.3.rs-3377247/v1)

**Keywords:** Epiretinal Membrane, Internal Limiting Membrane, Probe, Surgery, Treatment, Vitrectomy

## Abstract

This study describes a novel technique for removing the internal limiting membrane (ILM) using a 25-G vitrectomy probe during the epiretinal membrane (ERM)/ILM double peeling procedure. After standard 3-port 25-gauge pars plana vitrectomy, ERM is removed using membrane forceps and appropriate staining. An incidental ILM flap may occur while washing out the ILM-specific dye with the vitrectomy probe. To prevent this, we suggest not withdrawing the vitrectomy probe, but instead applying proximal and tangential aspiration force over the ILM flap, which may be extended and peeled off by gentle probe maneuvers along the retinal surface. The technique is described in three cases, with details, and shown in a video. This approach has several advantages. It may reduce the number of entries into the posterior segment during pars plana vitrectomy, preventing inadvertent retinal breaks. Furthermore, in cases with fragile ILMs that are hard to elongate with forceps, the active aspiration force of the vitrectomy probe might rapidly extend and form large flaps. We suggest applying this technique in scenarios involving ERM/ILM double peeling and double staining, where an incidental ILM flap is found after washing out the ILM-specific dye.

##  INTRODUCTION

Epiretinal membrane (ERM) is a frequent finding in the ophthalmic examination of individuals older than 50 and is often identified incidentally because many cases present with few or no symptoms.^[1]^ ERM formation can be idiopathic or secondary to various pathologies such as retinal vascular disorders, retinal tear/detachment, trauma, or surgical complications. It involves the fibrotic transformation of precursor cells and production of the extracellular matrix, which exerts traction forces on the internal limiting membrane (ILM) and the underlying retinal structures.^[2]^ Spontaneous resolution is unlikely, and surgical removal of the ERM remains the primary therapeutic option.^[3]^ ERM/ILM double peeling has been recommended and supported by several lines of evidence to minimize the recurrence rate of ERM by up to 75% (95% CI, 51% to 88%).^[4]^ The superiority of double peeling over ERM peeling alone is probably due to the complete removal of microscopic residual fibrotic cellular components on the retinal surface.^[4]^

Conventional ERM/ILM double peeling necessitates double staining—usually with triamcinolone acetonide (TA) and brilliant blue G (BBG) as ERM- and ILM-specific dyes, respectively—and the use of fine-membrane forceps to pinch and grasp the membranes over the retina.^[3]^ Minor adverse effects on retinal microstructure, morphology, and function may ensue from the sole removal of ILM; such effects are often confined to subclinical levels.^[4]^ However, damage to the retinal surface during forceps manipulation may cause localized inner retinal disruption and even full-thickness retinal breaks.^[5]^ Iatrogenic damage to the retina, including punctate retinal hemorrhages and focal edema, has also been reported in the literature.^[6,7]^ Multiple entries of instruments into the posterior segment carry the risk of inadvertent peripheral retinal breaks. In our surgical experience for years, we have encountered surgical scenarios in which we could limit the use of membrane forceps in such cases to minimize the risk profile and operation time. In this report, we describe our experience on ILM rhexis using conventional 25-G vitrectomy probes.

##  SURGICAL METHOD

After standard 3-port 25-gauge pars plana vitrectomy, ERM is removed using membrane forceps and, as usual, TA assistance. After the underlying ILM is re-stained using BBG and the dye is washed out by the vitrectomy probe, it is possible to find a tiny, incidental ILM flap at the primary grasping site, where the ERM was first pinched. Of note, in our experience, free ILM flaps can also occur in any other area of the posterior pole following ERM removal (as in case No. 1). In this scenario, we suggest not withdrawing the vitrectomy probe; instead, proximal and tangential aspiration forces should be applied to the visible ILM flap with a suggested aspiration pressure of 150-200 mmHg. The developed ILM flap can be extended and peeled by delicate probe maneuvers over the retinal surface.

##  RESULTS

### Case 1

**Figure 1:**
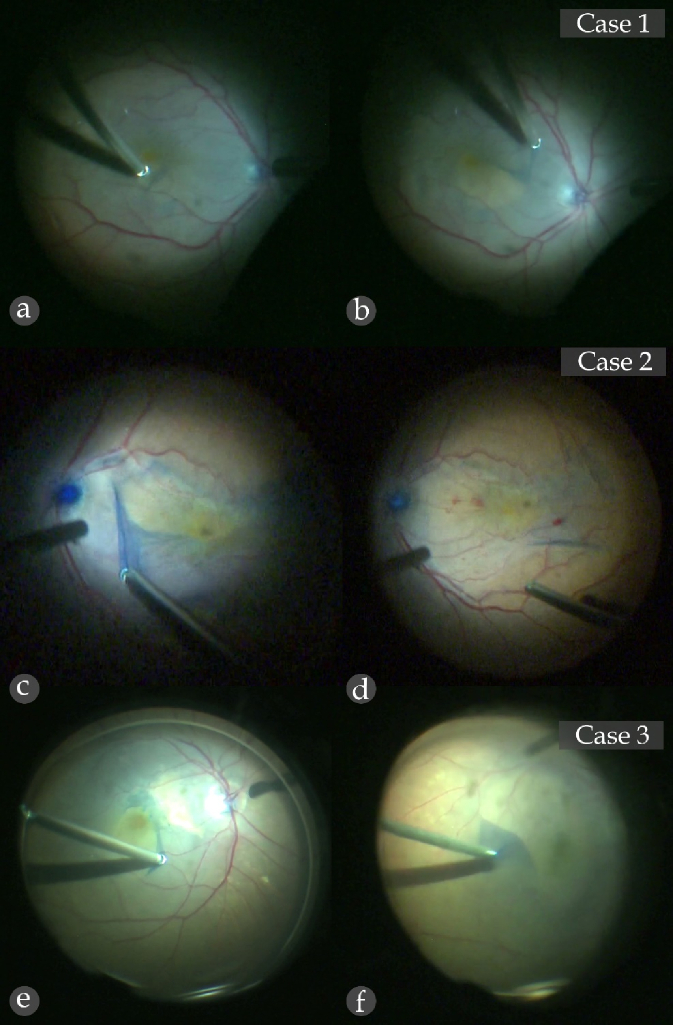
A brief demonstration of notable maneuvers in cases No. 1–3. (a & b) Case no. 1: (a) Noticing a tiny spontaneously-formed internal limiting membrane (ILM) flap in case No 1 and (b) peeling-off and extending the flap shown in part A. (c & d) Case no. 2: (c) Elongation of the flap through aspiration in case No. 2 and (d) applying aspiration force one disc diameter away from the flap lifts it off instantly and facilitates its grasping. (e & f) Case no. 3: (e) Grasping the ILM flap using the aspiration force of the probe in case no. 3 and (f) the maneuvers that cause circular curvilinear ILM-rhexis.

Video 1 (part 1) and Figure 1 (a & b) summarize the surgical process for a 59-year-old male patient with visually-impairing ERM. As shown, the ERM/posterior hyaloid face is first stained with TA and peeled by membrane forceps. After staining with BBG, a tiny ILM flap, seemingly unrelated to the primary pinch site, is found in the perifoveal region. The flap is elongated circularly to the desirable extent and removed. The patient's presurgical BCVA was 20/100, which improved to 20/70 after surgery.

### Case 2

As displayed in Video 1 (part 2) and Figure 1 (c & d), an extensive fibrotic ERM is being peeled off the retinal surface. Following the washout of BBG, a fold-like, large ILM flap is observed at the posterior pole. The flap is readily grasped and extended by the aspiration forces of the vitrectomy probe. The patient's preoperative BCVA was 20/100, which improved to 20/40 postoperatively.

### Case 3

Video 1 (part 3) and Figure 1 (e & f) show the operation process of a 69-year-old patient with combined ERM and macular lamellar hole. The ERM is grasped at the parafoveal region and removed. After washout of BBG, an ILM flap is detected along the primary direction of ILM peel-off. The maneuvers that cause circular curvilinear ILM-rhexis are notable. Patient's BCVA remained unaffected, as it measured 3/10 both before and after surgery.

### Notes

As shown in Video 1, the constructed ILM flaps can be extended and adjusted to the desired form and degree. As seen in Figure 1 (d), applying an aspiration force from about one disc diameter away from the ILM flap can lift it off instantly and facilitate grasping.

Video 1: Presentation of the probe-assisted ILM-rhexis in three cases.

##  DISCUSSION

To our knowledge, this surgical maneuver has not been described in the literature. This technique is preferable in cases where the purpose is to remove both the ILM and ERM. In such scenarios, we have frequently encountered situations where the ERM is initially removed and the peeling site is restained.

During the washout of BBG dye using the vitrectomy probe, one may find a free tiny flap of ILM at the primary ILM grasping site or elsewhere. In such cases, the surgeon would have two choices. The first (conventional) option is to withdraw the vitrectomy probe, reinsert the forceps, and proceed with ILM peeling. The second (novel) option is to continue with the probe and grasp the flap with mere aspiration. This attempt may lead to an immediate and easy peel-off of the ILM and its elongation to the desired extent.

The primary advantage of this technique is that reducing the number of entries into the posterior segment during pars plana vitrectomy will prevent the formation of inadvertent breaks posterior to the sclerotomy sites. Furthermore, based on the authors' experience, in fragile ILMs that are hard to be elongated by forceps, the active aspiration force of the vitrectomy probe can rapidly extend and form large flaps, possibly minimizing flap loss in these cases. Based on our experience, using the probe for membrane removal rather than forceps shortens surgical duration. Although no complication was encountered with using the probe, the safety profile of this method, compared to forceps grasping of the retinal tissue, requires further investigation.

It is important to note that this technique is not recommended in cases of neurosensory retinal detachment or retinoschisis, due to the significantly higher risk of retinal injuries. In eyes with fragile ILM, an uncontrolled suction force of the probe might result in flap tears, requiring surgeons to apply greater precision while utilizing this technique.

Overall, we have applied this maneuver in numerous cases where ERM/ILM double peeling was planned. The promising results we have noted with this technique compared to the conventional approach justify future research.

##  Financial Support and Sponsorship

None.

##  Conflicts of Interest

None.

## References

[1] Díaz-Valverde A, Wu L (2018). To peel or not to peel the internal limiting membrane in idiopathic epiretinal membranes. Retina.

[2] Fung AT, Galvin J, Tran T (2021). Epiretinal membrane: A review. Clin Exp Ophthalmol.

[3] Shimada H, Nakashizuka H, Hattori T, Mori R, Mizutani Y, Yuzawa M (2009). Double staining with brilliant blue G and double peeling for epiretinal membranes. Ophthalmology.

[4] Azuma K, Ueta T, Eguchi S, Aihara M (2017). Effects of internal limiting membrane peeling combined with removal of idiopathic epiretinal membrane: A systematic review of literature and meta-analysis. Retina.

[5] Rush RB, Simunovic MP, Aragon AV, Ysasaga JE (2014). Postoperative macular hole formation after vitrectomy with internal limiting membrane peeling for the treatment of epiretinal membrane. Retina.

[6] Asencio-Duran M, Manzano-Muñoz B, Vallejo-García JL, García-Martínez J (2015). Complications of macular peeling. J Ophthalmol.

[7] Oh HN, Lee JE, Kim HW, Yun IH (2013). Clinical outcomes of double staining and additional ILM peeling during ERM surgery. Korean J Ophthalmol.

